# 3-Bromo­propyl 2-(2-chloro­phen­yl)-2-(4,5,6,7-tetra­hydro­thieno[3,2-*c*]pyridin-5-yl)acetate

**DOI:** 10.1107/S1600536810053134

**Published:** 2010-12-24

**Authors:** Ji-Fang Chen, Ying Liu, Jing-Yang Wang, Deng-Ke Liu

**Affiliations:** a Materials Science and Engineering, Tianjin Polytechnic University, Tianjin 300160, People’s Republic of China; b Tianjin Institute of Pharmaceutical Research, Tianjin 300193, People’s Republic of China

## Abstract

In the crystal structure of the title compound, C_18_H_19_BrClNO_2_S, weak C—H⋯O inter­actions help to establish the packing.

## Related literature

The title compound is a derivative of the anti­platelet agent clopidogrel [systematic name (+)-(*S*)-methyl 2-(2-chloro­phen­yl)-2-(6,7-dihydro­thieno[3,2-*c*]pyridin-5(4*H*)-yl)acetate]. For background to the bioactivity and applications of clopidogrel, see: Muller *et al.* (2003 ▶); Savi *et al.* (1994 ▶); Sharis *et al.* (1998 ▶). For the synthesis of other derivatives with thienopyridine, see: Aubert *et al.* (1985 ▶); Bouisset & Radisson (1991 ▶); Savi *et al.* (1992 ▶); Bipin *et al.* (2002 ▶); Eric & Hiralal (1989 ▶); Liu *et al.* (2008 ▶); Silva (2004 ▶).
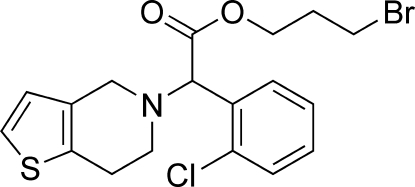


## Experimental

### 

#### Crystal data

C_18_H_19_BrClNO_2_S
*M* *_r_* = 428.76
Monoclinic, 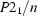

*a* = 8.5707 (17) Å
*b* = 18.414 (4) Å
*c* = 12.206 (2) Å
β = 106.89 (3)°
*V* = 1843.3 (7) Å^3^
*Z* = 4
Cu *K*α radiation
μ = 5.52 mm^−1^
*T* = 113 K
0.22 × 0.18 × 0.14 mm

#### Data collection

Rigaku Saturn diffractometer
Absorption correction: multi-scan (*CrystalClear*; Rigaku, 2005) *T*_min_ = 0.377, *T*_max_ = 0.512
18506 measured reflections
3546 independent reflections
3220 reflections with *I* > 2σ(*I*)
*R*_int_ = 0.055

#### Refinement

*R*[*F* ^2^ > 2σ(*F* ^2^)] = 0.035
*wR*(*F* ^2^) = 0.097
*S* = 1.04
3546 reflections
217 parameters
H-atom parameters constrained
Δρ_max_ = 0.34 e Å^−3^
Δρ_min_ = −0.69 e Å^−3^

### 

Data collection: *CrystalClear* (Rigaku/MSC, 2005) ▶; cell refinement: *CrystalClear* ▶; data reduction: *CrystalClear* ▶; program(s) used to solve structure: *SHELXS97* (Sheldrick, 2008 ▶); program(s) used to refine structure: *SHELXL97* (Sheldrick, 2008 ▶); molecular graphics: *SHELXTL* (Sheldrick, 2008 ▶); software used to prepare material for publication: *CrystalStructure* (Rigaku/MSC, 2005) ▶.

## Supplementary Material

Crystal structure: contains datablocks global, I. DOI: 10.1107/S1600536810053134/rk2241sup1.cif

Structure factors: contains datablocks I. DOI: 10.1107/S1600536810053134/rk2241Isup2.hkl

Additional supplementary materials:  crystallographic information; 3D view; checkCIF report

## Figures and Tables

**Table 1:** Hydrogen-bond geometry (Å, °)

*D*—H⋯*A*	*D*—H	H⋯*A*	*D*⋯*A*	*D*—H⋯*A*
C2—H2⋯O1^i^	0.95	2.43	3.340 (3)	161
C7—H7*A*⋯O1	0.99	2.50	3.103 (3)	119
C8—H8⋯Cl1	1.00	2.58	3.110 (2)	113
C16—H16*B*⋯Br1	0.99	2.91	3.323 (3)	106

Symmetry code: (i) 
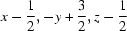
.

**Fig. 1.**
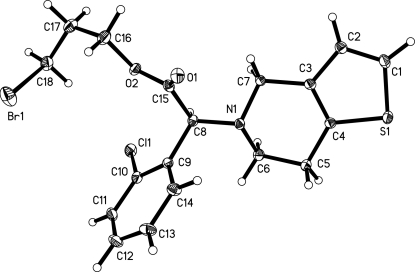
The molecular structure of title compound with the atom numbering scheme. Displacement ellipsoids are drawn at the 50% probability level. H atoms are presented as a small spheres of arbitrary radius.

## supplementary crystallographic information

### Comment

Clopidogrel is an oral, thienopyridine class antiplatelet agent used to inhibit blood clots in coronary artery disease, peripheral vascular disease, and cerebrovascular disease (Muller *et al.*, 2003; Aubert *et al.*, 1985; Bipin *et al.*, 2002; Bouisset & Radisson, 1991; Eric & Hiralal, 1989; Liu *et al.*, 2008; Silva 2004; Savi *et al.*, 1992; Savi *et al.*, 1994; Sharis *et al.*, 1998). The molecular structure of the title compound (Fig. 1), a derivative of clopidogrel, is reported here.

As shown in Fig. 1, there is a chiral carbon (C8) in the compound, and the benzene ring, the ester chain and the thienopyridine group are all linked to C8 and a molecular chiral center is formed. The thiophene ring of the thienopyridine group forms a plane and the C9–C14 benzen ring forms another plane. The dihedral angle formed between them is 65.46 (9)°. The packing molecules in crystal is consolidated by weak C—H···O, C—H···Cl and C—H···Br interactions.

### Experimental

The title compound was prepared according to the literature (Aubert *et al.*, 1985). A mixture of α–bromo(2–chloro)phenyl acetic acid (5 g, 20 mmol), 3–bromo–1–propanol (20 g, 144 mmol) and *p*–toluenesulfonic acid (1.0 g, 5.8 mmol) in toluene (50 ml) was refluxed for 2 h. The reaction mixture was washed with saturated sodium bicarbonate (100 ml) and then with distilled water (50 ml), dried with sodium sulfate and evaporated, to give colourless oil (98% yield). The colourless oil obtained above, K_2_CO_3_ (36 mmol) and 4,5,6,7–tetrahydro thieno[3,2–*c*] pyridin (22 mmol) in toluene (50 ml) were stirred at room temperature for 3 h. After removing the insoluble solid by filtration, the filtrate was concentrated and separated by flash chromatography to provide the target compound (yield 87%). Colourless single crystals were grown from a solution of petroleum ether and ethyl acetate (1:1 *v*/*v*).

### Refinement

All the H atoms were positioned geometrically and refined as riding atoms, with C—H = 1.00Å for methine, 0.99Å for methylene and C—H = 0.95Å for the other groups with *U*_iso_(H) = 1.2*U*_eq_(C).

### Crystal data

**Table:**

C_18_H_19_BrClNO_2_S	*F*(000) = 872
*M**_r_* = 428.76	*D*_x_ = 1.545 Mg m^−^^3^
Monoclinic, *P*2_1_/*n*	Cu *K*α radiation, λ = 1.54187 Å
Hall symbol: -P 2yn	Cell parameters from 2390 reflections
*a* = 8.5707 (17) Å	θ = 27.5–72.3°
*b* = 18.414 (4) Å	µ = 5.52 mm^−^^1^
*c* = 12.206 (2) Å	*T* = 113 K
β = 106.89 (3)°	Prism, colourless
*V* = 1843.3 (7) Å^3^	0.22 × 0.18 × 0.14 mm
*Z* = 4	

### Data collection

**Table:**

Rigaku Saturn diffractometer	3546 independent reflections
Radiation source: fine-focus sealed tube	3220 reflections with *I* > 2σ(*I*)
multilayer	*R*_int_ = 0.055
Detector resolution: 14.63 pixels mm^-1^	θ_max_ = 72.5°, θ_min_ = 4.5°
ω scans	*h* = −10→10
Absorption correction: multi-scan (*CrystalClear*; Rigaku, 2005)	*k* = −22→22
*T*_min_ = 0.377, *T*_max_ = 0.512	*l* = −14→11
18506 measured reflections	

### Refinement

**Table:**

Refinement on *F*^2^	Primary atom site location: structure-invariant direct methods
Least-squares matrix: full	Secondary atom site location: difference Fourier map
*R*[*F*^2^ > 2σ(*F*^2^)] = 0.035	Hydrogen site location: inferred from neighbouring sites
*wR*(*F*^2^) = 0.097	H-atom parameters constrained
*S* = 1.04	*w* = 1/[σ^2^(*F*_o_^2^) + (0.0543*P*)^2^ + 1.1251*P*] where *P* = (*F*_o_^2^ + 2*F*_c_^2^)/3
3546 reflections	(Δ/σ)_max_ = 0.001
217 parameters	Δρ_max_ = 0.34 e Å^−^^3^
0 restraints	Δρ_min_ = −0.69 e Å^−^^3^

### Special details

**Table:**

Geometry. All s.u.'s (except the s.u. in the dihedral angle between two l.s. planes) are estimated using the full covariance matrix. The cell s.u.'s are taken into account individually in the estimation of s.u.'s in distances, angles and torsion angles; correlations between s.u.'s in cell parameters are only used when they are defined by crystal symmetry. An approximate (isotropic) treatment of cell s.u.'s is used for estimating s.u.'s involving l.s. planes.
Refinement. Refinement of *F*^2^ against ALL reflections. The weighted *R*–factor *wR* and goodness of fit *S* are based on *F*^2^, conventional *R*–factors *R* are based on *F*, with *F* set to zero for negative *F*^2^. The threshold expression of *F*^2^ > σ(*F*^2^) is used only for calculating *R*–factors(gt) *etc*. and is not relevant to the choice of reflections for refinement. *R*–factors based on *F*^2^ are statistically about twice as large as those based on *F*, and *R*–factors based on ALL data will be even larger.

### Fractional atomic coordinates and isotropic or equivalent isotropic displacement parameters (Å^2^)

**Table:**

	*x*	*y*	*z*	*U*_iso_*/*U*_eq_	
Br1	0.70621 (4)	0.800075 (19)	0.63501 (3)	0.04494 (13)	
Cl1	0.63184 (7)	0.55788 (3)	0.47473 (5)	0.02681 (15)	
S1	0.70637 (7)	0.51762 (3)	−0.19215 (5)	0.02410 (14)	
O1	0.7341 (2)	0.75560 (8)	0.22244 (14)	0.0255 (3)	
O2	0.57129 (19)	0.72271 (8)	0.32887 (14)	0.0229 (3)	
N1	0.7310 (2)	0.60648 (9)	0.15415 (15)	0.0160 (3)	
C1	0.5719 (3)	0.58635 (13)	−0.2481 (2)	0.0247 (5)	
H1	0.5313	0.5965	−0.3276	0.030*	
C2	0.5302 (3)	0.62460 (12)	−0.16565 (19)	0.0215 (4)	
H2	0.4566	0.6644	−0.1807	0.026*	
C3	0.6102 (2)	0.59768 (11)	−0.05354 (18)	0.0173 (4)	
C4	0.7094 (3)	0.54013 (11)	−0.05401 (18)	0.0186 (4)	
C5	0.8122 (3)	0.50420 (12)	0.05215 (19)	0.0212 (4)	
H5A	0.9276	0.5188	0.0665	0.025*	
H5B	0.8052	0.4508	0.0430	0.025*	
C6	0.7515 (3)	0.52699 (11)	0.15262 (18)	0.0190 (4)	
H6A	0.6459	0.5030	0.1464	0.023*	
H6B	0.8306	0.5112	0.2252	0.023*	
C7	0.5925 (3)	0.62862 (11)	0.05604 (18)	0.0190 (4)	
H7A	0.5879	0.6823	0.0509	0.023*	
H7B	0.4892	0.6113	0.0679	0.023*	
C8	0.6982 (2)	0.62768 (11)	0.26063 (17)	0.0168 (4)	
H8	0.5998	0.6012	0.2680	0.020*	
C9	0.8421 (3)	0.61285 (11)	0.36541 (18)	0.0181 (4)	
C10	0.8233 (3)	0.58555 (11)	0.46718 (19)	0.0205 (4)	
C11	0.9545 (3)	0.57802 (12)	0.5653 (2)	0.0263 (5)	
H11	0.9384	0.5594	0.6338	0.032*	
C12	1.1084 (3)	0.59798 (13)	0.5619 (2)	0.0300 (5)	
H12	1.1986	0.5940	0.6287	0.036*	
C13	1.1313 (3)	0.62372 (14)	0.4615 (2)	0.0310 (5)	
H13	1.2378	0.6360	0.4589	0.037*	
C14	0.9995 (3)	0.63170 (12)	0.3643 (2)	0.0245 (5)	
H14	1.0166	0.6503	0.2961	0.029*	
C15	0.6710 (3)	0.70946 (11)	0.26505 (19)	0.0185 (4)	
C16	0.5484 (4)	0.79869 (12)	0.3521 (2)	0.0314 (6)	
H16A	0.4978	0.8250	0.2798	0.038*	
H16B	0.6545	0.8217	0.3907	0.038*	
C17	0.4393 (3)	0.80103 (14)	0.4279 (2)	0.0340 (6)	
H17A	0.3316	0.7811	0.3849	0.041*	
H17B	0.4228	0.8525	0.4455	0.041*	
C18	0.4994 (3)	0.76028 (14)	0.5389 (2)	0.0302 (5)	
H18A	0.5145	0.7085	0.5227	0.036*	
H18B	0.4165	0.7631	0.5809	0.036*	

### Atomic displacement parameters (Å^2^)

**Table:**

	*U*^11^	*U*^22^	*U*^33^	*U*^12^	*U*^13^	*U*^23^
Br1	0.02728 (18)	0.0748 (3)	0.03292 (19)	−0.01154 (13)	0.00900 (13)	−0.00602 (13)
Cl1	0.0272 (3)	0.0339 (3)	0.0236 (3)	−0.0020 (2)	0.0142 (2)	0.0024 (2)
S1	0.0222 (3)	0.0319 (3)	0.0195 (3)	0.0020 (2)	0.0081 (2)	−0.0041 (2)
O1	0.0295 (9)	0.0216 (7)	0.0257 (8)	−0.0053 (6)	0.0086 (7)	−0.0009 (6)
O2	0.0255 (8)	0.0222 (7)	0.0237 (8)	0.0041 (6)	0.0116 (7)	−0.0012 (6)
N1	0.0140 (8)	0.0195 (8)	0.0148 (9)	−0.0004 (6)	0.0047 (7)	−0.0018 (6)
C1	0.0228 (11)	0.0338 (12)	0.0172 (11)	−0.0044 (9)	0.0051 (9)	−0.0004 (9)
C2	0.0176 (11)	0.0251 (10)	0.0203 (11)	0.0000 (8)	0.0029 (8)	0.0013 (8)
C3	0.0130 (10)	0.0218 (10)	0.0173 (10)	−0.0031 (8)	0.0045 (8)	−0.0007 (8)
C4	0.0153 (10)	0.0225 (10)	0.0185 (10)	−0.0015 (8)	0.0059 (8)	−0.0014 (8)
C5	0.0195 (11)	0.0246 (10)	0.0196 (11)	0.0047 (8)	0.0059 (9)	−0.0009 (8)
C6	0.0179 (10)	0.0196 (10)	0.0189 (11)	0.0018 (8)	0.0047 (8)	0.0008 (8)
C7	0.0163 (10)	0.0234 (10)	0.0167 (10)	0.0024 (8)	0.0038 (8)	−0.0010 (8)
C8	0.0148 (10)	0.0201 (10)	0.0163 (10)	−0.0036 (7)	0.0058 (8)	−0.0014 (8)
C9	0.0178 (10)	0.0180 (9)	0.0176 (11)	−0.0003 (8)	0.0036 (8)	−0.0026 (7)
C10	0.0232 (11)	0.0203 (10)	0.0192 (11)	−0.0017 (8)	0.0081 (9)	−0.0031 (8)
C11	0.0326 (13)	0.0258 (11)	0.0175 (11)	0.0011 (9)	0.0025 (9)	−0.0016 (8)
C12	0.0286 (13)	0.0293 (12)	0.0245 (12)	0.0009 (10)	−0.0042 (10)	−0.0032 (9)
C13	0.0186 (12)	0.0335 (12)	0.0363 (14)	−0.0035 (9)	0.0007 (10)	−0.0006 (10)
C14	0.0189 (11)	0.0291 (11)	0.0252 (12)	−0.0042 (9)	0.0058 (9)	0.0020 (9)
C15	0.0152 (10)	0.0215 (10)	0.0163 (10)	−0.0006 (8)	0.0008 (8)	−0.0022 (8)
C16	0.0432 (16)	0.0220 (11)	0.0302 (14)	0.0103 (10)	0.0128 (12)	−0.0002 (9)
C17	0.0275 (13)	0.0368 (13)	0.0380 (15)	0.0113 (10)	0.0102 (11)	−0.0063 (11)
C18	0.0208 (12)	0.0403 (13)	0.0321 (13)	−0.0035 (10)	0.0118 (10)	−0.0084 (10)

### Geometric parameters (Å, °)

**Table:**

Br1—C18	1.961 (3)	C7—H7B	0.9900
Cl1—C10	1.746 (2)	C8—C9	1.522 (3)
S1—C1	1.714 (2)	C8—C15	1.527 (3)
S1—C4	1.729 (2)	C8—H8	1.0000
O1—C15	1.204 (3)	C9—C10	1.392 (3)
O2—C15	1.335 (3)	C9—C14	1.397 (3)
O2—C16	1.452 (3)	C10—C11	1.391 (3)
N1—C8	1.461 (3)	C11—C12	1.382 (4)
N1—C6	1.475 (3)	C11—H11	0.9500
N1—C7	1.478 (3)	C12—C13	1.380 (4)
C1—C2	1.358 (3)	C12—H12	0.9500
C1—H1	0.9500	C13—C14	1.388 (3)
C2—C3	1.429 (3)	C13—H13	0.9500
C2—H2	0.9500	C14—H14	0.9500
C3—C4	1.359 (3)	C16—C17	1.495 (4)
C3—C7	1.502 (3)	C16—H16A	0.9900
C4—C5	1.494 (3)	C16—H16B	0.9900
C5—C6	1.524 (3)	C17—C18	1.503 (4)
C5—H5A	0.9900	C17—H17A	0.9900
C5—H5B	0.9900	C17—H17B	0.9900
C6—H6A	0.9900	C18—H18A	0.9900
C6—H6B	0.9900	C18—H18B	0.9900
C7—H7A	0.9900		
			
C1—S1—C4	91.66 (11)	C15—C8—H8	109.6
C15—O2—C16	115.84 (18)	C10—C9—C14	117.4 (2)
C8—N1—C6	109.32 (16)	C10—C9—C8	122.62 (19)
C8—N1—C7	109.27 (16)	C14—C9—C8	119.78 (19)
C6—N1—C7	109.52 (16)	C9—C10—C11	122.0 (2)
C2—C1—S1	112.29 (17)	C9—C10—Cl1	120.45 (17)
C2—C1—H1	123.9	C11—C10—Cl1	117.54 (18)
S1—C1—H1	123.9	C12—C11—C10	119.2 (2)
C1—C2—C3	111.9 (2)	C12—C11—H11	120.4
C1—C2—H2	124.1	C10—C11—H11	120.4
C3—C2—H2	124.1	C13—C12—C11	120.1 (2)
C4—C3—C2	113.13 (19)	C13—C12—H12	119.9
C4—C3—C7	121.65 (19)	C11—C12—H12	119.9
C2—C3—C7	125.21 (19)	C12—C13—C14	120.3 (2)
C3—C4—C5	123.63 (19)	C12—C13—H13	119.8
C3—C4—S1	111.03 (16)	C14—C13—H13	119.8
C5—C4—S1	125.28 (16)	C13—C14—C9	120.9 (2)
C4—C5—C6	108.83 (17)	C13—C14—H14	119.5
C4—C5—H5A	109.9	C9—C14—H14	119.5
C6—C5—H5A	109.9	O1—C15—O2	124.55 (19)
C4—C5—H5B	109.9	O1—C15—C8	126.0 (2)
C6—C5—H5B	109.9	O2—C15—C8	109.33 (17)
H5A—C5—H5B	108.3	O2—C16—C17	107.0 (2)
N1—C6—C5	110.68 (17)	O2—C16—H16A	110.3
N1—C6—H6A	109.5	C17—C16—H16A	110.3
C5—C6—H6A	109.5	O2—C16—H16B	110.3
N1—C6—H6B	109.5	C17—C16—H16B	110.3
C5—C6—H6B	109.5	H16A—C16—H16B	108.6
H6A—C6—H6B	108.1	C16—C17—C18	115.6 (2)
N1—C7—C3	110.59 (17)	C16—C17—H17A	108.4
N1—C7—H7A	109.5	C18—C17—H17A	108.4
C3—C7—H7A	109.5	C16—C17—H17B	108.4
N1—C7—H7B	109.5	C18—C17—H17B	108.4
C3—C7—H7B	109.5	H17A—C17—H17B	107.4
H7A—C7—H7B	108.1	C17—C18—Br1	111.47 (18)
N1—C8—C9	112.41 (17)	C17—C18—H18A	109.3
N1—C8—C15	111.46 (17)	Br1—C18—H18A	109.3
C9—C8—C15	104.01 (16)	C17—C18—H18B	109.3
N1—C8—H8	109.6	Br1—C18—H18B	109.3
C9—C8—H8	109.6	H18A—C18—H18B	108.0
			
C4—S1—C1—C2	−0.33 (18)	C15—C8—C9—C10	99.5 (2)
S1—C1—C2—C3	0.3 (2)	N1—C8—C9—C14	45.0 (3)
C1—C2—C3—C4	−0.1 (3)	C15—C8—C9—C14	−75.8 (2)
C1—C2—C3—C7	179.0 (2)	C14—C9—C10—C11	1.0 (3)
C2—C3—C4—C5	177.21 (19)	C8—C9—C10—C11	−174.4 (2)
C7—C3—C4—C5	−2.0 (3)	C14—C9—C10—Cl1	−177.92 (16)
C2—C3—C4—S1	−0.1 (2)	C8—C9—C10—Cl1	6.7 (3)
C7—C3—C4—S1	−179.26 (15)	C9—C10—C11—C12	−0.2 (3)
C1—S1—C4—C3	0.24 (17)	Cl1—C10—C11—C12	178.70 (18)
C1—S1—C4—C5	−177.02 (19)	C10—C11—C12—C13	−1.2 (4)
C3—C4—C5—C6	16.2 (3)	C11—C12—C13—C14	1.9 (4)
S1—C4—C5—C6	−166.92 (16)	C12—C13—C14—C9	−1.1 (4)
C8—N1—C6—C5	−171.04 (17)	C10—C9—C14—C13	−0.3 (3)
C7—N1—C6—C5	69.3 (2)	C8—C9—C14—C13	175.2 (2)
C4—C5—C6—N1	−48.6 (2)	C16—O2—C15—O1	−3.8 (3)
C8—N1—C7—C3	−171.30 (16)	C16—O2—C15—C8	173.21 (19)
C6—N1—C7—C3	−51.6 (2)	N1—C8—C15—O1	−32.0 (3)
C4—C3—C7—N1	19.3 (3)	C9—C8—C15—O1	89.4 (3)
C2—C3—C7—N1	−159.78 (19)	N1—C8—C15—O2	151.02 (17)
C6—N1—C8—C9	65.3 (2)	C9—C8—C15—O2	−87.6 (2)
C7—N1—C8—C9	−174.82 (16)	C15—O2—C16—C17	−177.7 (2)
C6—N1—C8—C15	−178.34 (17)	O2—C16—C17—C18	57.6 (3)
C7—N1—C8—C15	−58.5 (2)	C16—C17—C18—Br1	61.8 (3)
N1—C8—C9—C10	−139.74 (19)		

### Hydrogen-bond geometry (Å, °)

**Table:**

*D*—H···*A*	*D*—H	H···*A*	*D*···*A*	*D*—H···*A*
C2—H2···O1^i^	0.95	2.43	3.340 (3)	161.
C7—H7A···O1	0.99	2.50	3.103 (3)	119.
C8—H8···Cl1	1.00	2.58	3.110 (2)	113.
C16—H16B···Br1	0.99	2.91	3.323 (3)	106.

Symmetry codes: (i) *x*−1/2, −*y*+3/2, *z*−1/2.
